# Birds of a Feather Flock Together: Disadvantageous Decision Making in Augmented Restless Legs Syndrome Patients with and without Impulse Control Disorders

**DOI:** 10.3390/brainsci11030383

**Published:** 2021-03-17

**Authors:** Beatrice Heim, Philipp Ellmerer, Ambra Stefani, Anna Heidbreder, Elisabeth Brandauer, Birgit Högl, Klaus Seppi, Atbin Djamshidian

**Affiliations:** Department of Neurology, Medical University of Innsbruck, 6020 Innsbruck, Austria; beatrice.heim@i-med.ac.at (B.H.); philipp.ellmerer@tirol-kliniken.at (P.E.); ambra.stefani@i-med.ac.at (A.S.); anna.heidbreder@i-med.ac.at (A.H.); elisabeth.brandauer@tirol-kliniken.at (E.B.); birgit.ho@i-med.ac.at (B.H.); klaus.seppi@i-med.ac.at (K.S.)

**Keywords:** restless legs syndrome, behavioral abnormalities, impulsivity, decision making

## Abstract

Background: Augmentation (AUG) in patients with restless legs syndrome (RLS) can be associated with impulse control disorder (ICD) symptoms, such as compulsive sexual behavior, gambling disorder or compulsive shopping. In this study, we wanted to assess whether RLS patients with AUG differ in decision making from those patients who have augmentation and in addition ICD symptoms (AUG + ICD) in a post hoc analysis of a patient cohort assessed in a previous study. Methods: In total, 40 RLS patients with augmentation (19 AUG + ICD, 21 AUG without ICDs) were included. RLS diagnosis, severity, and diagnosis of augmentation were made by sleep disorder specialists. ICD symptoms were assessed using semi-structured interviews. All patients performed the beads task, which is an information sampling task where participants must decide from which of the two cups colored beads were drawn. Results were compared to 21 healthy controls (HC). Results: There was no difference in information sampling or irrational decision making between AUG and AUG + ICD patients (*p* = 0.67 and *p* = 1.00, respectively). Both patient groups drew less beads and made more irrational decisions than HC (all *p*-values < 0.03, respectively). Conclusions: Our results suggest that augmentation itself is associated with poorer decision making even in the absence of ICD symptoms. Further studies are necessary to explore whether rapid and hasty decision making are a harbinger of augmentation in RLS.

## 1. Introduction

Restless legs syndrome (RLS) is a common sensorimotor disorder affecting up to 10% of the European and North American population [1,2], and is characterized by unpleasant sensations of the limbs during rest often accompanied by sleep complaints [3]. The pathogenesis is still not fully understood, but several studies have shown that pathophysiological mechanisms including central iron dyshomeostasis and dopaminergic dysfunction are mainly involved [4,5]. 

Dopaminergic medication can, however, trigger augmentation (AUG) as well as symptoms of impulse control disorders (ICDs) [6,7]. In fact, RLS patients with augmentation have an almost 6-fold increased risk of exhibiting ICD symptoms compared to RLS patients without augmentation [8]. Both, patients with AUG as well as those with ICD symptoms have an impairment in decision making [9] and may therefore share a common pathomechanism [8]. Previous studies showed that drug naïve RLS patients [10] as well as those treated with dopaminergic medication [9] jump to conclusions and make more decisions against the evidence on the beads task than HC. Interestingly, RLS patients with augmentation decide more often irrational than HC and RLS patients without augmentation [9]. Due to the common coexistence of ICD symptoms and AUG, it is unclear whether jumping to conclusion behavior is triggered by the addictive behavior or whether AUG alone can cause impairment in information sampling.

Therefore, we aimed to explore the role of the presence of ICD symptoms in decision making in these RLS patients with augmentation in a post hoc analysis of the same cohort.

## 2. Methods

RLS diagnosis and diagnosis of augmentation were made by sleep disorder specialists in 40 RLS patients with augmentation at the Department of Neurology of the Medical University of Innsbruck, as described earlier [9]. RLS symptom severity was assessed using the IRLS scale. ICD symptoms were assessed using semi structural interviews based on the questionnaire for impulsive compulsive behavior in Parkinson’s disease (QUIP) [11] and Levodopa equivalent units (LEU) were calculated, as described previously [9]. We excluded patients with other diseases possibly aggravating RLS symptoms, e.g., renal dysfunction. Moreover, participants with severe neuropsychiatric disorders, e.g., major depression, or neuroleptic drug intake, were excluded. Furthermore, we compared results with 21 HC, recruited as described previously [9]. Augmentation was categorized into mild and severe symptoms as recommended [12]. We included 19 patients with augmentation and ICD symptoms (AUG + ICD), 21 RLS patients with augmentation only (AUG), and 21 HC.

All patients performed the beads task, an information sampling task to assess rash and/or irrational decisions [13], which has shown to be sensitive to detect early impairments in decision making in other neurodegenerative and psychiatric disorders like Parkinson’s disease [14], Huntington’s disease [15], substance abuse [16], as well as RLS as described previously [9,10]. In the beads task, participants have to guess from which of two cups colored beads are drawn. The beads task was performed as described previously [9]. The final goal is to make as many correct guesses as possible (detailed task descriptions are found elsewhere [14].

## 3. Statistics

Statistical analyses were performed using SPSS 23.0 (IBM, Armonk, NY, USA). Parametric and non-parametric tests as well as the Fisher’s Exact test were used for statistical analysis depending on the distribution and the scale type of the variables. To test for normality, the Kolmogorov–Smirnov test was used. Data were analyzed with parametric statistics where normality assumptions were met. Otherwise, non-parametric tests were used. Outcome measures in this task are number of beads drawn prior to decide in each ratio (“drawing behavior”), and number of decisions against the evidence (“opposite color choice”, e.g., blue bead shown, green cup chosen). A generalized linear model was used with either the number of draws before making a decision or the number of decisions that were contrary to the evidence participants had at that time (i.e., irrational decision making) as dependent variables. A Poisson model which had a log-linear link function was used. All pairwise comparisons were Bonferroni corrected. A *p*-value below 0.05 (2-sided) was considered to indicate statistical significance.

## 4. Results

Demographic and clinical data of the RLS and HCs are described in detail in a former publication [9]. There was a significant group difference between the three groups (AUG, AUG + ICDs, HC) in drawing behavior (*p* < 0.001) and irrational decision making (*p* = 0.008) (Table 1).

**Figure 1:**
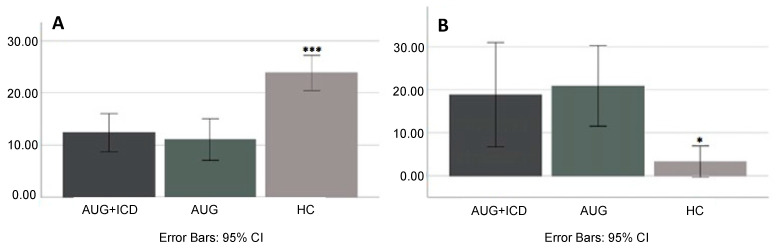
(**A**) average beads drawn per group; (**B**) average irrational decisions made per group. Error bars are +/− SE, 95% Cl. Significant differences are labelled with “*”; * *p* < 0.05; *** *p* < 0.001. AUG, RLS patients with augmentation only; AUG + ICD, augmented RLS patients with ICD symptoms; HC, healthy controls.

Post hoc analysis showed no difference in information sampling (*p* = 0.67) or illogical decision making (*p* = 1.00) between AUG and AUG + ICD patients, but we found significant differences between the patient groups and HC: both patient groups gathered significantly less evidence prior to making a decision than HC (both *p*-values < 0.001); moreover, both patient groups made more irrational decisions than HC (*p* < 0.001 and *p* < 0.03, respectively) (Table 1, Figure 1A,B).

## 5. Discussion

In this post hoc analysis, we showed that RLS patients with augmentation seek less evidence and make more irrational decisions than HC, irrespective of whether ICD symptoms were present or not. Functional imaging studies have demonstrated that in the beads task the selection of a cup, rather than drawing more evidence, correlated with larger hemodynamic responses in the striatum and the anterior cingulate [17], likely representing a higher dopamine release in these structures. Dopamine agonists are known to reduce the interaction of the striatum to the prefrontal cortex [18] and at the same time increase ventral striatal activity [18,19,20]. It is unclear whether augmented RLS patients have deficits in fronto-striatal top-down inhibition (“top down control”) or have an increase impulsive drive due to excessive striatal dopamine levels (“bottom up impulsivity”) or a combination of both. It is likely that impulsive choices, as demonstrated by the beads task, are due to neuroplastic changes caused by long-term dopaminergic medication similar to what has been described in patients with Parkinson’s disease and ICDs [14,21]. One study on multimodal MR imaging in RLS identified higher connectivity within the left executive network, associated with attention, working memory, and decision making [22]. Interestingly, in our study additional presence of an ICD symptom did not worsen performance on the beads task. In line with this, poorer performance on the frontal assessment battery (FAB) test was found in RLS patients with augmentation [23], suggesting again impairment in executive dysfunction, which is similar to what was found previously in patients with Parkinson’s disease with ICD [24]. In our cohort, there was no group difference between augmented RLS patients with and without ICD symptoms in LEU dose or IRLS. Moreover, Bayard et al. reported that RLS patients, irrespective of taking dopaminergic medication or drug free, showed preferences toward risky choices on the Iowa Gambling Task [25]. These findings are comparable to a former study [10], which showed that reflection impulsivity is common in RLS patients, regardless whether they are drug naïve or treated with dopaminergic therapy. These results suggest that making disadvantageous choices may be a disease-specific behavioral abnormality in RLS patients, which might be further impaired but not solely explained by dopaminergic therapy. Thus, our findings presented here further strengthen the hypothesis that augmentation and ICD symptoms may share a similar pathomechanism which then either present as augmentation, ICD symptoms, or frequently a combination of both. Nevertheless, further studies are needed to confirm our hypothesis and to subsequent characterize risk factors for developing augmentation and non-motor symptoms in RLS.

## Figures and Tables

**Table 1:** Clinical and demographic characteristics.

	AUG + ICD	AUG	HC	*p*-Value
Number (*n*)	19	21	21	
Mild augmentation (*n* = 26)	9	17	-	0.026 *
Severe augmentation (*n* = 14)	10	4		
ICD symptoms	19	-	-	-
Gender (male:female)	9:9	8:14	7:14	0.73
Age (years)	61.6 ± 12.0	66.6 ± 7.6	59.5 ± 11.1	0.098
Education (years)	11.0 ± 3.1	10.1 ± 1.8	11.1 ± 1.6	0.46
Disease duration (years)	19.9 ± 16.0	16.3 ± 13.1	-	0.15
IRLS (at time of assessment)	25.3 ± 7.8	24.2 ± 7.5	-	1.0
LEU (mg)	153.1 ± 180.4	109.1 ± 133.1	-	0.81
DA monotherapy (all)	15	15	-	-
Pramipexole	11	12		
Rotigotine	3	2		
Ropinirole	1	1		
Levodopa monotherapy	2	3	-	-
Levodopa plus DA	1	4 *	-	-
Pramipexole	-	3		
Rotigotine	1	2		
Ropinirole	-	1		
Total draws (*n*) ± SD ^a^	12.4 ± 15.7	11.1 ± 18.1	22.9 ± 16.0	<0.001 ***
Irrational choices (*n*) ± SD ^b^	2.0 ± 2.4	2.0 ± 2.1	0.3 ± 0.8	0.008 **

* one patient had Levodopa + pramipexole + rotigotine. Significant differences are labelled with “*”; * *p* < 0.05; ** *p* < 0.01; *** *p* < 0.001. ^a^ AUG + ICD vs. AUG, *p* = 0.67; AUG + ICD vs. HC, *p* < 0.001 ***; AUG vs. HC, *p* < 0.001 ***. ^b^ AUG + ICD vs. AUG, *p* = 1.0; AUG + ICD vs. HC, *p* = 0.022 *; AUG vs. HC, *p* = 0.018 *. AUG + ICD, augmented RLS patients with ICD symptoms; AUG, RLS patients with augmentation only; DA, dopamine agonist; IRLS, International RLS Study Group Rating Scale; LEU, levodopa equivalent units; *n*, number; SD, standard deviation.

## Data Availability

Data is contained within the article.
